# No Apparent Increase in Treatment Uptake for Gambling Disorder during Ten Months of the COVID-19 Pandemic—Analysis of a Regional Specialized Treatment Unit in Sweden

**DOI:** 10.3390/ijerph18041918

**Published:** 2021-02-17

**Authors:** Anders Håkansson, Gunny Åkesson, Cécile Grudet, Niroshani Broman

**Affiliations:** 1 Psychiatry, Department of Clinical Sciences Lund, Faculty of Medicine, Lund University, 221 85 Lund, Sweden; cecile.grudet@med.lu.se (C.G.); niroshani.broman@med.lu.se (N.B.); 2 Gambling Disorder Unit, Malmö Addiction Center, Region Skåne, 205 02 Malmö, Sweden; gunny.akesson@skane.se

**Keywords:** gambling disorder, problem gambling, treatment seeking, COVID-19, pandemic

## Abstract

The COVID-19 pandemic and its impact on society has been suspected to affect gambling behaviors. Potentially, the pandemic crisis may result in increased problem gambling, for example, due to COVID-19-related psychological distress, unemployment, and financial difficulties. In addition, the cancellation of sports in early parts of the crisis has been suspected to alter gambling behaviors. Policy makers have called for action and, in some cases, have changed regulations, and media have reported possible increases in treatment seeking. However, research data are hitherto lacking. The present study assessed the treatment uptake at a regional specialized gambling-disorder unit in the healthcare system of Region Skåne, Sweden. Number of patients, treatment contacts, and patterns of rescheduling or cancellations of appointments were quantified for each month, January–December 2020, and compared to corresponding months of 2018 and 2019. Possible trends were analyzed, using an interrupted time-series analysis. Results did not indicate an increase in treatment uptake for gambling disorder during the months of COVID-19 impact in Sweden. The proportion of digital treatment increased, but total treatment uptake was unaffected by the pandemic. In conclusion, during the first ten months of the pandemic in Sweden, no obvious increase in treatment uptake for gambling disorder could be seen. Moreover, longer follow-up may be necessary in order to see if effects of worsening socioeconomic conditions may be a possible long-term risk factor of increased gambling after COVID-19.

## 1. Introduction

The spread of SARS-CoV-2 virus, and the COVID-19 disease affecting humanity worldwide, has been shown to have substantial health impact beyond acute infectious disease. Mental health consequences have been cited among the many health hazards following the pandemic [1,2]. Among these, it has been suggested that addictive behaviors, including problem gambling, may be altered or potentially enhanced. Such an alteration could be due to life-style changes during COVID-19-related restriction in everyday life or a consequence of worsening psychological health in the population [3,4]. Concerning problem gambling, increased time spent online, altered working conditions, unemployment, financial stress, or poor mental health have all been suggested as possible drivers of a COVID-19-related increase in problem gambling [5]. Thus, the pandemic may theoretically have an impact also on the prevalence or course of gambling disorder, a condition affecting patients and their concerned significant others dramatically, including both social and psychological consequences [6].

Early in the pandemic, in several countries, political and academic stakeholders raised issues related to a potential need to prevent problem gambling during the pandemic. These initiatives included, for example, a partial ban on gambling advertising in Spain [7], a deposit limit introduced in the United Kingdom [8], and reduced online gambling in Belgium. Moreover, a total ban against some types of gambling was settled in Latvia, and Sweden introduced a deposit limit and other measures intended to lower risk in online casino gambling and in land-based electronic-gambling-machine gambling [9]. Studies carried out during 2020 suggest that overall changes to gambling behavior because of COVID-19 may be modest in the general adult population [10]. However, there may be reason to suspect some transition between gambling types, since specific venues and/or certain types of gambling were closed down or reduced [11,12,13,14]. Two European observational studies of actual gambling behavior in online gambling operators have shown modest changes in the gambling market. These studies demonstrated none [15] or limited [16] transition of gambling from sports betting to online casino betting, in early phases of the pandemic. One early finding of an adult web survey was that individuals reporting increased gambling during COVID-19 were more likely to be problem gamblers prior to the pandemic, as well [10]. This leads to the assumption that potential negative consequences in this area may primarily affect people already at risk.

In the early debate, fears of an extensive impact of the pandemic on gambling behaviors included potentially increased treatment needs for problem gambling, which, anecdotally, was reported by peer-support organizations, in the beginning. However, systematic data on the issue are largely unavailable [17,18]. Conversely, due to the COVID-19 circumstances, there was also a concern for decreased treatment seeking. For example, such a possible decrease could be due to home confinement, fears of being exposed to the virus at treatment facilities, and possible changes in provided services. Such changes have been reported to occur in other types of treatment facilities for addictive disorders [19,20,21]. Therefore, there is a need for research studying whether pandemic-related changes in gambling may have translated into an increased—or potentially decreased—uptake of treatment for gambling disorder. To the best of the authors’ knowledge, no such study data on actual treatment uptake for gambling problems during COVID-19 have been made available so far.

Based on previously reported changes in gambling behavior, specifically in problem gamblers, and media reports expressing the same fear, the study hypothesized that an increase would be seen in the treatment uptake for gambling disorder during COVID-19. Thus, the theoretical model, based on the fears expressed by policy makers and researchers, suggesting possibly increasing gambling problems in the society [5,6,7,8,9,10], is that the COVID-19 period may demonstrate higher treatment uptake in a specialized treatment unit aimed for patients with gambling disorder. Thus, based on the research gap described above, the present study aimed to assess treatment uptake in a regional specialized treatment unit for adult patients with gambling disorder, month by month, throughout a large period of time during the COVID-19 pandemic in Sweden. Data were compared to the corresponding time periods of the past two years.

## 2. Materials and Methods

Region Skåne is the healthcare organization of the county of Skåne, in Southern Sweden, a county with around 1.3 million inhabitants. The Gambling Disorder Unit is a part of the addiction center of the county’s urban center, Malmö, but has a regional responsibility and admits patients (aged 18 years and above) with gambling disorder from the whole region. Treatment provided includes medical psychiatric assessment and individual (and on rare occasions group) therapy for gambling disorder, and the treatment is covered, to a large extent, by public tax funding. In a previous retrospective study, 58 percent of treated patients were documented to be diagnosed with secondary psychiatric diagnoses apart from the gambling disorder [22].

The facility was opened in December 2015, in an era when gambling disorder was still not recognized as a condition to include in the treatment obligations of Swedish healthcare providers or in social services [23]. In January 2018, gambling disorder was introduced in the legislation, stating the obligations of Swedish healthcare and municipal social services in January 2018. Since January 2018, no other formal changes have been made to the treatment uptake regulations in the region. In January 2019, a new Swedish gambling act introduced a nationwide self-exclusion service [24], which attracted substantial attention by the media.

In the present setting, the most commonly displayed gambling types in television advertising promote rapid online-based gambling types. Among these gambling types, the online casino is by far the most common [25]. In the treatment facility studied here, a large majority of treatment-seeking patients report online gambling, with online-casino gambling being most prominent [22]. Problem gambling, measured in general population surveys, has been reported to occur in around two percent of the adult population [26]. The legal gambling age in Sweden is 18 years, which is also typically the age limit for treatment uptake in adult addiction psychiatry.

As a certain degree of seasonality can be suspected in treatment uptake, only months available to study during the index year (2020) were included from the preceding years. With respect to changes over the year, a lower staff coverage happens from late June to early August, which is most pronounced during July, due to summer holidays.

In the present study, data of registered visits and contacts at the unit were extracted from the regional healthcare databases of the overall healthcare organization, Region Skåne, by a financial manager of the psychiatric department of Malmö who has a high degree of experience in digitalized systems or hospital management and follow-up. Data were extracted from each of the months of 2020, in order to study the potential effects of different phases of the pandemic, as well as the time period immediately prior to it. For comparison, data from the same months were extracted for 2019 and 2018, i.e., data covering the full months of January through December 2020, 2019, and 2018. Thus, throughout the whole period analyzed here, the same legislation regarding treatment responsibility was in use.

In Sweden, the month of March, in 2020, can be considered the first month in which the COVID-19 pandemic had a major impact on society. In mid-March, a number of crisis reports appeared in the media, and a number of government restrictions and crisis plans were decided. For example, government recommendations to work at home and to close high schools and universities were issued on March 16 and 17, respectively. Sports-betting markets, worldwide, experienced a considerable perturbation, as major soccer leagues were cancelled gradually, from early March until late March. On April 1, Swedish state-owned land-based casinos were entirely closed (and remained closed when the present analyses were carried out). Government restrictions on gambling, announced on April 23, were in effect from July 2. After a considerable decrease in COVID-19 cases during the summer, a considerable surge in COVID-19 cases was seen in October 2020, and maintained to an increasing degree in November and December, resulting in new government restrictions.

Data include the following information: number of unique patients registered for an assessment or treatment contact during one month, number of such contacts during one month (regardless of whether these occurred in unique patients or on repeated occasions), proportion of women and men among the unique patients, and the distribution of primary diagnoses of unique patients during one month. In addition, the number of cancellations and changes of appointments, as well as the number of occasions where a patient did not show up for an appointment, was documented. Moreover, the proportion of distance appointments by telephone or video was documented. As the primary diagnosis was, in the vast majority of cases, the gambling disorder, with or without psychiatric secondary diagnoses, no further analyses of diagnoses were carried out in the present study.

The study was sent for ethical review by the Swedish Ethics Review Board (file number 2020-03232). However, the Swedish Ethics Review Board decided that the present type of study did not require ethical permission according to Swedish law, as no identified personal data were treated. Moreover, for the same reason, no information or informed consent procedure was carried out. In order to protect confidentiality of patients and avoiding the risk of too few individuals meeting a certain criterion during one single month, the study did not report detailed data such as age or reporting of diagnoses correlated with age or gender.

### Statistical Methods

Data were analyzed by using an interrupted-time-series analysis, for each of the outcome measures (numbers of patients, number of total contacts, number of female and male patients, number of changed/cancelled appointments, and number or no-shows. Here, independent variables in the analyses were time (monthly) and COVID-19 (the COVID-19-affected period was set to begin with the month of March 2020, and COVID-19-unaffected months were all months prior to that). In addition, a Student’s *t*-test was run, comparing the mean monthly number of treatment contacts, number of changes/cancellations, and numbers of no-shows, after vs. prior to the onset of COVID-19. Associations with a *p*-value of <0.05 were considered significant. For statistical calculations, the software IBM SPSS version 25.0 was used.

## 3. Results

**Figure 1:**
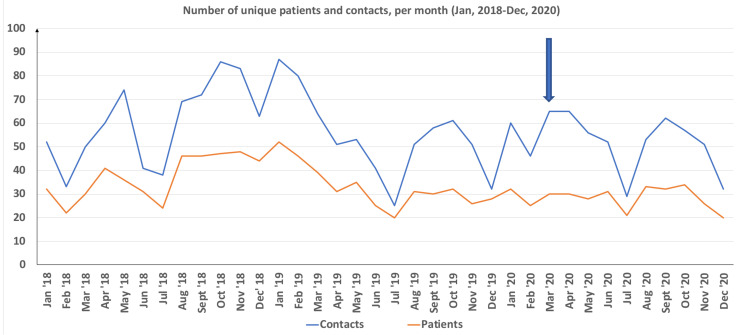
Number of unique patients and number of treatment contacts per month.

Table 1 displays, for each month studied, the number of unique patients, the total number of contacts, the proportion of women among unique patients (Figure 1), the proportion of treatment contacts carried out digitally, the total number of cancellations/changes of appointments, and the total number of no-shows.

In interrupted time series analyses, the number of unique patients seen (*p* = 0.72), the number of unique female (*p* = 0.43) and male (*p* = 0.97) patients, the total number of contacts (*p* = 0.65), and the number of no-shows (*p* = 0.49) were not significantly affected by the COVID-19 pandemic. The number of changes/cancellations, however, displayed a significant decline (*p* < 0.01) related to COVID-19. For each of these outcome measures, no significant effect of the overall time variable was seen for the whole study period, except for a marginally significant decrease in total treatment contacts (*p* = 0.06) over time. While distance contacts were low or non-existent for the majority of months prior to the pandemic, they increased steeply from March 2020 (*p* < 0.001).

Using the Student’s *t*-test, we saw that there was no significant COVID-19-related difference in number of monthly contacts (52.6 vs. 57.0 prior to the pandemic, *t*(23) = 0.87, *p* = 0.39). Moreover, no difference was seen with respect to the number of no-shows (6.3 vs. 7.9 prior to the pandemic, *t*(23) = 1.46, *p* = 0.16). During the pandemic, the monthly number of changes/cancellations was significantly lower (14.3 vs. 22.9, *t*(18) = 3.92, *p* = 0.001).

## 4. Discussion

The present study analyzed treatment uptake in a specialized gambling-disorder unit during the COVID-19 pandemic, in comparison to the two preceding years. The study aimed to contribute to the understanding of a potential impact of the pandemic on gambling behaviors in the society, as discussed in media reporting of a potential surge in gambling-related treatment seeking during the pandemic. The present study was carried out in a system where similar units are rare, and thereby, a limited number of patients were analyzed. However, despite the limited size of the facility, the study was able to demonstrate that the hypothesis of increased treatment uptake during COVID-19 was hitherto not supported.

It could be argued that a change in gambling behaviors in the population may not readily translate into actual treatment seeking in patients with a diagnostic level of problem gambling. However, the present unit has a well-established treatment service for such disorder, which has been available throughout the pandemic as well as during the preceding years. Therefore, it can be suspected that awareness of the unit in peer-support organizations, social authorities in the area, and other potential sources of referral, such as primary care, was relatively well developed, already, at study start. Therefore, an absence of treatment uptake increase during 2020 is unlikely caused by changes in access to the unit or by limited awareness by other stakeholders about the existence of the unit. Altogether, this strengthens the fact that a COVID-19-related surge in treatment seeking during the study period would have been visible in the data presented here.

However, it can be argued that a potential change in gambling habits during COVID-19 would not cause an increase in treatment uptake as early on in the pandemic as the months studied here. The last month included in the study was December 2020, which may be considered as relatively early within the pandemic. December 2020 is, at most, ten months after the pandemic became extensively discussed in the general public in Sweden and seven to nine months after the most extensive perturbations of the gambling market due to the pandemic. However, beyond the potential change in gambling markets in early phases of COVID-19, more prolonged financial and social consequences have additionally been suspected to increase problem gambling over time. Likely, such effects would require a longer study period, to appear in treatment settings. In addition, the present study includes both the extensive viral transmission period early in the COVID-19 pandemic, as well as the surge in virus transmission during the autumn of 2020. New studies with a longer follow-up period are needed in order to explore such potential changes further. Moreover, it should be kept in mind that many people with gambling disorder do not seek treatment because of a number of actual or perceived barriers. Such barriers may include stigma and feelings of shame and guilt, or a wish to cope with one’s problems outside of the treatment system [27]. Thus, a limited treatment-seeking behavior for problem gambling should be accounted for in broader analyses of the treatment uptake during COVID-19. In contrast, it is unclear whether the decision-making in problem gamblers considering treatment seeking, based on resistance to seek treatment or feelings of stigma and shame, would differ during the pandemic, as compared to other times. Further, and likely, more in-depth and qualitative studies may be needed in order to highlight this.

In summary, the findings of the present study did not confirm the fear of increased treatment needs expressed in media reports and in the rationale behind policy changes. This adds to the previous data published from online surveys and market data, which were, however, analyzed early during the pandemic. An online survey showed that problem gamblers were considerably more likely to report having increased gambling during the pandemic [10]. In contrast, an overall increase in gambling in market data was not seen [16]. Thus, longer-term analyses of the gambling market activity were not available and cannot be compared to the treatment-seeking data studied here.

However, as the self-reported increase in gambling was seen specifically in problem gamblers in a previous study, an increase in treatment seeking was hypothesized here. Fear of attending physical treatment units has been cited as a barrier to treatment seeking in other addictive disorders [19,20,21] and may represent a factor that decreases the number of patients or masks a potential increase. From the present real-world data, describing a clinical context post hoc, it is clear that distance treatment—on telephone or digital video solutions—represented a large share of contacts taken with the clinic from March 2020 to December 2020, and it is very likely that the treatment uptake would have been lower without the possibility of distance treatment.

Thus, it is possible that our results could indicate a clear decrease in treatment activity, rather than only the absence of an increase. Therefore, it is clear that digital treatment plays a crucial role in gambling disorder treatment during COVID-19. As indicated in the previous literature, addiction treatment settings may face a number of challenges during the pandemic [20]. In addition, the quality and structure of digital contacts is likely hard to evaluate. While beyond the aims of the present study, the necessity of such adaptations is obvious. Moreover, the number of cancelled or rescheduled visits and no-shows by patients were not obviously high during the pandemic-affected months; no-shows were not significantly affected by the pandemic, and changes and cancellations rather decreased, as opposed to the opposite, during the pandemic. Possibly, the option of digital contacts may have facilitated contacts in many ways and, thus, active treatment could continue despite potential fears of visiting the facility. However, given the possibility of distance contacts, the absence of an increased treatment uptake further confirms that an obvious increase in treatment seeking hitherto has not occurred. In addition to the study findings reported here, future studies also need to assess changes in mood and gambling-related craving in patients already treated. Based on the reports of self-reported gambling increase in problem gamblers, it could be suspected that patients admitted to the unit may display a more severe clinical picture. Various factors may affect problem gamblers in different directions. For example, lifestyle changes, financial crisis, and unemployment may increase the severity of gambling [5]. In contrast, it has also been reported that the distinct periods of society lockdown may instead be relieving to problems gamblers, as specific gambling opportunities are reduced [28]. While this goes beyond the present study, these potential changes may need to be explored further in future studies.

It could be argued that a highly specialized unit is probably not the arena most likely to detect an early change in gambling behaviors. The work load of such a unit depends on the problem severity of patients referred there and by the presence of patients with severe problems who remain in treatment for an extended period of time. Thus, again, the present type of data, well within the core of treatment services for individuals with severe gambling problems, need to be merged with data from sources covering individuals with less severe gambling problems or individuals caught earlier in their development of a gambling problem. These sources of data may, for example, include the use of responsible gambling tools offered by gambling operators or help-line calls. It is also well-known that individuals seeking help for gambling problems may do so in a broad range of ways, where contact outside of the specialized treatment system may represent a viable option to many individuals [29]. In preliminary data from the national Swedish help line for gambling problems, previously reported in a publicly available oral presentation, the total number of contacts to the help line had decreased during the pandemic period, as compared to 2019. A pronounced decrease in help calls was seen during the first weeks of the most prominent COVID-19-related impact on sports-betting markets [30]. This is in line with the data presented here, where an obvious influx of treatment seekers during COVID-19 cannot be seen to date.

In addition, it should be kept in mind that the present study setting, Sweden, is one of the countries applying a less restrictive policy with respect to COVID-19 transmission; policy measures such as lockdown, stay-at-home orders, and closing of schools or work places have been absent or limited, in contrast to many others countries [31,32,33].

The present study may have implications for stakeholders in the addiction-treatment area and could be a starting point for further research. It may serve as a baseline analysis to future, more long-term studies of the evolvement of treatment seeking during the pandemic. Moreover, it strengthens the reasons to continue following gambling behaviors during COVID-19 with several different methodologies: specialized treatment seeking, actual measures of gambling behavior, and perceptions of gambling problems in the population, since they are likely to represent different sides of the story. A potential increase in gambling problems within certain groups may not automatically be visible as a change in special-unit treatment seeking. Moreover, vice versa, the findings in the present study may neither confirm nor exclude a change in gambling behavior in the general population and demonstrate the need for further studies in the area.

Clearly, the present study has limitations, which are of value to address. The study was carried out in a single region-wide specialized unit. Few comparable units are available in the present setting, and, therefore, a broader nationwide analysis of such treatment units could not be carried out. Based on this fact, the number of included individuals is low, and the study is hence subject to a higher degree of statistical uncertainty. Moreover, as the study could not assess specific identified individuals, an individual patient may be counted in the data for more than one month; thus, figures from several months cannot be added to one another. This, however, means that the study reflects the work load and, thereby, general treatment uptake in a specialized gambling treatment unit, rather than the absolute number of new patients.

Moreover, in the present type of overview, it cannot be established whether problem severity or other characteristics of treatment queries may have differed over time, as only diagnostic data of primary diagnosis were available. Therefore, psychiatric comorbidity, known to be common in patients at this clinical facility [22], could not be studied. Another limitation is that, potentially, in the clinical everyday work, a treatment-seeking contact from a patient may not always result in an actual treatment episode at the unit, for example, if the contact is mainly a question about treatment possibilities or if the patient is decided to be better referred to a different type of unit. Such more informal queries made are difficult to quantify. However, it should be kept in mind that the routes for contact initiation by patients formally have been the same over the study period and that the staff involved in receiving these contacts also have been the same over time. Thus, while more informal contact attempts cannot be measured and therefore represent a limitation of the study, the extent of this limitation may be minor. It should also be noted that the present study assessed treatment uptake in adults, in a facility admitting patients from 18 years of age and above. Younger adults may be more likely to be problem gamblers than older people and, therefore, potentially represent a particular risk group [24]. However, treatment uptake is less distinctly defined for adolescents below 18 years of age. This group may not typically be seen in specialized gambling centers or in the healthcare systems; thus, those who are below the legal gambling age may have a different gambling pattern compare to adults. Based on comparable publications available thus far, by largely observing adults in the legal gambling market [12,15,16] or in the adult population [10,11], nothing is known about how gambling below legal gambling age is affected by COVID-19. Likewise, for minors, where voluntary treatment seeking may not occur under the same circumstances as for adults, treatment needs for problem gambling during the pandemic are hitherto unknown.

## 5. Conclusions

It can be concluded, from the present data in a gambling-disorder unit in Sweden, that, hitherto, no apparent increase in treatment seeking during COVID-19 could be seen, in contrast to media reports of possible increases in treatment-seeking behavior for gambling disorder. However, although this study assessed a region-wide unit, with a broad uptake of patients, it must be kept in mind that absolute numbers in the study are low. As gambling disorder is a condition with typically low rates of treatment seeking, more studies, in more than one setting, may be needed in order to assess larger numbers and gain greater statistical power. Moreover, changes in the society believed to cause increased gambling problems during the pandemic may be of a long-term nature. Thus, further studies and thorough observations are needed.

## Figures and Tables

**Table 1:** Number of patients involved in treatment for gambling disorder at the regional Gambling Disorder Unit, Malmö Addiction Center, Malmö, Sweden.

Month	Unique Patients, Total, n ^1^	Contacts, Total, n	Proportion of Women, n (%) ^1^	Proportion of Distance Contacts, %	Number of Changed or Cancelled Bookings (by Patient), n	Number of No-Shows, n
Jan. 2018	32	52	6 (19)	0	13	8
Feb. 2018	22	33	2 (9)	0	17	9
Mar. 2018	30	50	5 (17)	0	22	7
Apr. 2018	41	60	5 (12)	0	22	8
May 2018	36	74	6 (17)	2	38	9
June 2018	31	41	4 (13)	5	22	5
July 2018	24	38	4 (16)	16	14	7
Aug. 2018	46	69	12 (26)	10	20	6
Sept. 2018	46	72	10 (22)	7	23	16
Oct. 2018	47	86	9 (19)	2	31	10
Nov. 2018	48	83	13 (27)	1	27	15
Dec. 2018	44	63	14 (32)	2	17	9
Jan. 2019	52	87	16 (31)	3	26	14
Feb. 2019	46	80	13 (28)	5	29	8
Mar. 2019	39	64	14 (36)	0	31	11
Apr. 2019	31	51	12 (39)	4	22	3
May 2019	35	53	15 (43)	0	23	6
June 2019	25	41	9 (36)	0	13	4
July 2019	20	25	5 (25)	0	19	3
Aug. 2019	31	51	10 (32)	2	18	4
Sept. 2019	30	58	10 (33)	0	24	10
Oct. 2019	32	61	11 (34)	0	29	4
Nov. 2019	26	51	5 (19)	0	21	2
Dec. 2019	28	32	9 (32)	9	28	8
Jan. 2020	32	60	8 (25)	2	17	11
Feb. 2020	25	46	4 (16)	2	29	8
Mar. 2020	30	65	7 (23)	43	21	9
Apr. 2020	30	65	3 (10)	57	14	8
May 2020	28	56	4 (14)	52	13	3
June 2020	31	52	5 (16)	25	7	6
July 2020	21	29	4 (19)	3	19	2
Aug. 2020	33	53	5 (15)	11	25	4
Sept. 2020	32	62	4 (13)	18	12	7
Oct. 2020	34	57	8 (24)	9	14	7
Nov. 2020	26	51	5 (19)	20	9	7
Dec. 2020	21	36	4 (19)	56	9	10

^1^ Unique patients per month. For this variable, figures across months may be overlapping and figures for different months cannot be summarized.

## Data Availability

Data can be made available from the researcher upon request.
